# Increased Dicarbonyl Stress as a Novel Mechanism of Multi-Organ Failure in Critical Illness

**DOI:** 10.3390/ijms18020346

**Published:** 2017-02-07

**Authors:** Bas C. T. van Bussel, Marcel C. G. van de Poll, Casper G. Schalkwijk, Dennis C. J. J. Bergmans

**Affiliations:** 1 Department of Intensive Care, Maastricht University Medical Centre +, Maastricht 6229 HX, The Netherlands; marcel.vande.poll@mumc.nl (M.C.G.v.d.P.); d.bergmans@mumc.nl (D.C.J.J.B.); 2 Department of Surgery, and NUTRIM School for Nutrition and Translational Research, Maastricht University Medical Centre +, Maastricht 6229 HX, The Netherlands; 3 Department of Internal Medicine, and CARIM School for Cardiovascular Diseases, Maastricht University Medical Centre +, Maastricht 6229 HX, The Netherlands; c.schalkwijk@maastrichtuniversity.nl

**Keywords:** dicarbonyl stress, glyoxalase, methylglyoxal, critical care, multi-organ failure, persistent critical illness

## Abstract

Molecular pathological pathways leading to multi-organ failure in critical illness are progressively being unravelled. However, attempts to modulate these pathways have not yet improved the clinical outcome. Therefore, new targetable mechanisms should be investigated. We hypothesize that increased dicarbonyl stress is such a mechanism. Dicarbonyl stress is the accumulation of dicarbonyl metabolites (i.e., methylglyoxal, glyoxal, and 3-deoxyglucosone) that damages intracellular proteins, modifies extracellular matrix proteins, and alters plasma proteins. Increased dicarbonyl stress has been shown to impair the renal, cardiovascular, and central nervous system function, and possibly also the hepatic and respiratory function. In addition to hyperglycaemia, hypoxia and inflammation can cause increased dicarbonyl stress, and these conditions are prevalent in critical illness. Hypoxia and inflammation have been shown to drive the rapid intracellular accumulation of reactive dicarbonyls, i.e., through reduced glyoxalase-1 activity, which is the key enzyme in the dicarbonyl detoxification enzyme system. In critical illness, hypoxia and inflammation, with or without hyperglycaemia, could thus increase dicarbonyl stress in a way that might contribute to multi-organ failure. Thus, we hypothesize that increased dicarbonyl stress in critical illness, such as sepsis and major trauma, contributes to the development of multi-organ failure. This mechanism has the potential for new therapeutic intervention in critical care.

## 1. Introduction

Sepsis and major trauma often develop into multi-organ failure and persistent critical illness, which have a mortality rate of 20%–40% [1,2]. To date, the exact underlying pathobiology explaining how sepsis and major trauma cause multi-organ failure, and eventually evolve to persistent critical illness, remains incompletely understood [3,4]. Increased inflammation, impaired coagulation, endothelial dysfunction leading to microvascular dysfunction [4], and mitochondrial dysfunction leading to increased oxidative stress [5], appear to be involved in this process, but are unable to fully explain the observed multi-organ failure and persistent critical illness [3,6]. Importantly, trials in critical illness that aimed to decrease inflammation [7,8], restore coagulation [9], improve endothelial dysfunction [4], and reduce oxidative stress [10,11], did not improve survival rates [12]. Novel underlying potential mechanisms, linking increased inflammation, impaired coagulation, endothelial dysfunction, and increased oxidative stress, on the one hand, and multi-organ failure and persistent critical illness, on the other, to mortality, should be investigated. This may reveal new therapeutic targets which can be used in critical care.

We hypothesize that increased dicarbonyl stress in sepsis or major trauma contributes to the development of multi-organ failure and persistent critical illness, and is associated with increased mortality. Dicarbonyl stress is the abnormal intracellular accumulation of dicarbonyl metabolites [13]. The reactive dicarbonyls—i.e., methylglyoxal, glyoxal, and 3-deoxyglucosone—are produced by several metabolic pathways, such as anaerobic glycolysis, gluconeogenesis, and lipid peroxidation [14]. These dicarbonyls react with the amino groups of both intracellular and extracellular proteins, in a way that contributes to cell and tissue dysfunction [13,14,15]. Firstly, this process damages intracellular proteins, altering their function, which subsequently impairs cellular function. Secondly, this process also damages extracellular matrix components, which may affect tissue barrier function. Finally, it modifies plasma proteins that activate receptors on endothelial cells, mesangial cells, and macrophages, which induces the receptor-mediated production of reactive oxygen species and causes pathological changes in gene expression [13]. In particular, methylglyoxal has been suggested to play an important role in disease, as increased levels have been linked to diabetes, cardiovascular disease, cancer, and central nervous system disorders [16]. When considering health, methylglyoxal is detoxified by the glyoxalase system. The key enzyme of this major intracellular detoxification system is glyoxalase-1. In the presence of reduced glutathione, and the subsequent action of the enzyme glyoxalase-2, glyoxalase-1 detoxifies methylglyoxal into d-lactate [13,17]. This protects cells from dicarbonyl stress. Thus, increased metabolic stress and an impaired glyoxalase system, increases the dicarbonyl stress that impairs cellular function and interacts on multiple levels.

## 2. Dicarbonyl Stress in Disease States

In diabetes, the mechanism of increased dicarbonyl stress that causes protein modifications, is known as the glycation pathway. Hyperglycaemia induces excessive superoxide production, which partly inhibits the glycolytic enzyme glyceraldehyde-3-phosphate dehydrogenase, resulting in an increased glucose flux that drives the formation of intracellular dicarbonyls [13]. The dicarbonyls cause protein modifications called advanced glycation end products (AGEs). In contrast to the formation of AGEs by glucose, protein modifications through reactive dicarbonyls form very rapidly [13]. The formation of AGEs by dicarbonyl stress has been extensively studied, and it is becoming clear that this is one of the major pathways causing the hyperglycaemia-induced complications of diabetes [13]. In fact, in diabetes, it has been shown that dicarbonyl stress and its protein modifications are involved in complications contributing to macro- and microvascular-, neurological-, and renal disease, on multiple levels [13,18]. Thus, dicarbonyl stress affects many organs.

An investigation of dicarbonyl stress could play a role in further unravelling the pathobiology of multi-organ failure and persistent critical illness. Increased dicarbonyl stress may affect many organs [13]. Experimental data have shown that this mechanism impairs the renal, cardiovascular, and central nervous system function, at least partially independent of hyperglycaemia [19,20,21]. An in vivo model in non-diabetic mice showed that the knockout of glyoxalase-1, modifies glomerular proteins and oxidative stress in a way that leads to an impaired renal function [19]. It has been shown, using non-diabetic rats, that the administration of methylglyoxal caused endothelial dysfunction and severe degenerative changes in cutaneous vessels, suggesting an impaired microcirculatory function [20]. In addition, methylglyoxal increases inflammation, leading to endothelial cell loss that contributes to diabetic cardiomyopathy [22]. The intraperitoneal infusion of methylglyoxal in C57BL/6 mice showed a neuroinflammatory response in astrocytes and the hippocampus, suggesting a methylglyoxal-induced impairment of the central nervous system function [21]. Whether dicarbonyl stress contributes to hepatic or respiratory failure, requires further investigation. Additionally, dicarbonyl stress has been involved in increased inflammation [15], impaired coagulation [23], endothelial dysfunction [24], and oxidative stress [14,25]. Each of these mechanisms appears to play a role in sepsis- and trauma-related multi-organ failure and persistent critical illness [3,4,5,6]. Thus, increased dicarbonyl stress could contribute to multi-organ failure in critical illness.

In critical illness, both hypoxia and inflammation, which are prevalent [3], as well as increased glucose metabolism, could increase dicarbonyl stress [26]. Firstly, hyperglycaemia likely plays a role as it often accompanies acute critical illness and the intracellular formation of dicarbonyl stress is rapid [13,17]. Indeed, an in vivo model of critical illness showed that hyperglycaemia-induced mitochondrial dysfunction causing liver and myocardial damage, was accompanied by elevated levels of methylglyoxal, glyoxal, and 3-deoxyglucosone [27]. However, important recent data have shown that monocytes, upon stimulation, switch their internal metabolism from oxidative phosphorylation, to glycolysis, in order to mount an increasingly effective response upon re-stimulation [28]. Although hypothetical, this metabolic switch to glycolysis likely increases the production of methylglyoxal, glyoxal, and 3-deoxyglucosone. However, such an increased production could be an adequate host response upon infection, and this is supported by recent data showing that methylglyoxal synthase is absent in Group A Streptococcus, with a glyoxalase system in place to detoxify methylglyoxal from external sources, such as activated host neutrophils [29]. Secondly, hypoxia and inflammation have been shown to increase methylglyoxal through the downregulation of glyoxalase activity, both in vitro and in vivo, independently from hyperglycaemia [15,30].

**Figure 1:**
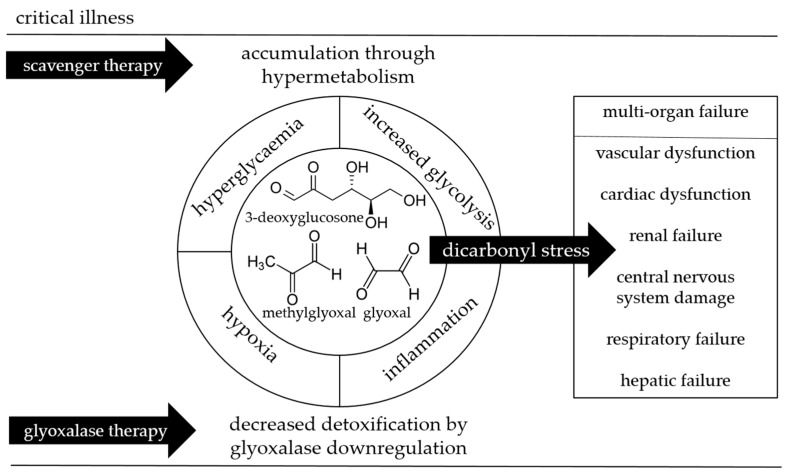
A schematic illustration of the hypothesis of whether increased dicarbonyl stress in critical illness contributes to the development of multi-organ failure. Dicarbonyl stress has been shown to impair renal function [19], cardiovascular function [20,22], and central nervous system function [21]. Whether it contributes to hepatic or respiratory function, requires further investigation. The inner circle shows methylglyoxal, glyoxal, and 3-deoxyglucosone, with their proposed determinants in critical illness in the outer circle, and indicated by arrows are scavenger therapy and glyoxalase therapy, as potential therapeutic targets.

Therefore, we hypothesize that hypoxia, inflammation, and increased glucose metabolism in critical illness, increase dicarbonyl stress; on the one hand, through the accumulation of methylglyoxal, glyoxal, and 3-deoxyglucosone through hypermetabolism, and on the other hand, through decreased detoxification by the downregulation of glyoxalase activity. These mechanisms thereby cause cellular dysfunction at multiple levels, that may lead to multi-organ failure and persistent critical illness associated with higher mortality (Figure 1). To draw firm conclusions, studying this hypothesis in critical care requires several considerations, particularly due to the fact that previous laboratory experiments exhibiting promising results, did not lead to novel therapeutics in critical care [4,7,8,9,10,11,12]. These considerations will be discussed below.

## 3. Potential Therapeutic Targets

So far, it has been demonstrated that methylglyoxal, and one of its protein modifications, *N*^ε^-(Carboxyethyl)lysine (CEL), were elevated in human sepsis and critical illness [31,32]. In addition, higher levels of the glyoxal-derived protein modification *N*^ε^-(Carboxymethyl)lysine (CML) have been associated with in-hospital mortality in critical illness, and both higher levels of CEL and CML have been associated with a higher SOFA score [32], which indicates a more severe disease [33]. No data are available for assessing whether protein modifications contribute to the long-term functional disability observed in intensive care unit survivors [34].

Although not much is known about the development of dicarbonyl stress in multi-organ failure or persistent critical illness, it may have potential consequences for the timing of promising therapeutic interventions. On the one hand, it is conceivable that the peak level of dicarbonyl stress, or the total amount during a specific time period after the onset of acute critical illness, drives the severity of multi-organ failure and persistent critical illness. On the other hand, a persistent exposure might contribute towards this even more significantly. Methylglyoxal plasma levels have been shown to be higher in individuals with septic shock, than in controls [31]. The levels peak at 24 h after the onset of sepsis, and then gradually decrease. Methylglyoxal-derived protein modifications show a similar pattern [31]. Recent data on methylglyoxal- and glyoxal-derived protein modifications in critical illness, measured by mass spectrometry analytical techniques, showed higher levels at the start of severe critical illness, versus control subjects, without a clear direction in the development over the following seven days [32]. These data may suggest that dicarbonyl stress develops very rapidly, before any apparent critical disease. Alternatively, these data may suggest that pre-existing comorbidities/conditions drive both an increase in dicarbonyl stress, and an increased likelihood of developing a critical disease, independent of each other. Repeated measurements of dicarbonyl stress in critical illness will provide more direction on this matter.

Reducing the accumulation of methylglyoxal, and enhancing glyoxalase activity, are among the potential therapeutic targets. Compounds with potential therapeutic actions have not yet been investigated in critical illness. However, low arginine plasma levels in intensive care unit patients with persistent septic or cardiogenic shock, relative to asymmetric dimethylarginine levels, have been associated with multi-organ failure and mortality [35]. This association might be explained by increased dicarbonyl stress, as plasma arginine quenches methylglyoxal [17]. However, this obviously warrants further study. Another promising compound that potentially lowers methylglyoxal, is pyridoxamine [17]. Furthermore, in vitro studies have shown that flavonoids, in particular quercetin, scavenge methylglyoxal [36,37]. In vivo, however, no data, to our knowledge, are available, and the low bioavailability of quercetin might hamper effects [38]. Aminoguanidine and alagebrium chloride also scavenge methylglyoxal [39]. Although trials with aminoguanidine and alagebrium chloride in patients with diabetes were terminated, due to an unfavourable perceived risk-to-benefit ratio, this ratio could be different in critical care [40]. Upregulation of the glyoxalase system, via stimulation of the nuclear factor erythroid 2-related factor 2 (Nrf2) by isothiocyanates, including sulforaphane, may reduce methylglyoxal in critical illness [41]. Of particular interest is a recent randomized study of healthy overweight and obese individuals, which showed that a combination of trans-resveratrol and hesperetin, synergized to increase glyoxalase-1 expression, and decreased plasma methylglyoxal and whole-body methylglyoxal-protein modification [42]. Finally, avoiding early hyperglycaemia in acute critical illness, as an additional target, prevents dicarbonyl stress [13], particularly as the critical time window for dicarbonyl stress exposure with regard to multi-organ failure, remains unknown. Notably, it is unlikely that mild glucose control, which appeared to prevent death in contrast to tight or very mild control in critical care [43], significantly contributes to dicarbonyl stress. Thus, although the optimal timing of any intervention is unclear, several potential compounds are promising for targeting dicarbonyl stress in critical care.

## 4. Biomarker Determination

To date, it is unknown which dicarbonyl or protein modification is most important in critical illness, although methylglyoxal is promising, as it appears to play an important role in disease [16]. Plasma methylglyoxal and one of its protein modifications, CEL, have been elevated in sepsis and critical illness [31,32], but only plasma glyoxal-derived CML has been associated with in-hospital mortality in critical illness, in an exploratory study [32]. Methylglyoxal, glyoxal, and 3-deoxyglucosone form many distinct protein modifications, such as methylglyoxal-hydroimidazolone-1, argpyrimidine, and pentosidine, amongst others, that, to our knowledge, have not yet been investigated in critical illness [13]. d-lactate, the methylglyoxal detoxification product [13,17], could mark the efficiency of the glyoxalase system in critical illness. However, in patients with septic shock, mucosal barrier failure provokes d-lactate absorption from the intestine, which hampers the use of d-lactate as a marker for methylglyoxal detoxification [44]. Acute glutathione depletion, for example, due to oxidative stress, has been shown to increase hepatic methylglyoxal levels [45], which suggests that glutathione should also be taken into account when investigating the role of methylglyoxal and the glyoxalase system in critical illness. Thus, since population-based data in critical illness which link dicarbonyls stress with outcome remain scarce, biomarker selection should be based on biochemical arguments, starting with the measurement of methylglyoxal, glyoxal, and 3-deoxyglucosone.

Another issue that requires attention is the body compartment where dicarbonyl stress should be investigated in critical illness. Higher plasma levels of CEL, CML, and pentosidine have been associated with incident cardiovascular disease [18], suggesting that plasma is an adequate reflection of body dicarbonyl stress. Although plasma levels of dicarbonyls are promising, their association with increased mortality is yet to be established. The local accumulation of methylglyoxal in carotid plaques has been associated with more plaque ruptures, which suggests a local effect [30]. In critical illness, the organ that dysfunctions the most might have the highest production of dicarbonyl stress, such as the lungs in acute respiratory distress syndrome. Bronchial alveolar lavage fluid, for example, could be investigated in pneumosepsis, and ascites/peritoneal punctate could be studied in patients with multi-organ failure, due to hepatic failure, necrotizing pancreatitis, or complicated abdominal surgery. Considered together, although plasma levels are promising, other compartments of local dicarbonyl stress, if possible, should also be explored in critical illness.

Importantly, measurements of dicarbonyls or protein modifications require analytical techniques that are valid and reproducible, as shown for methylglyoxal, glyoxal, and 3-deoxyglucosone, using ultra performance liquid chromatography tandem-mass spectrometry [46]. Skin autofluorescence, another indirect marker of dicarbonyl stress, which measures the fluorescence of modified proteins, has been shown to be reproducible and elevated in critical illness [32]. However, skin autofluorescence was not associated with markers of disease severity or mortality in critical illness [32].

## 5. Comorbidities, Medication, and Other Sources of Dicarbonyl Stress

Medication use, such as metformin, ACE inhibitors, angiotensin receptor inhibitors, statins, or anti-TNF therapy, has been suggested to possibly lower dicarbonyl stress [40]. Thiamine, often administered in critical care because of assumed deficiency, diverts glucose metabolism from glycolysis through the activation of transketolase, and thereby may reduce methylglyoxal [39]. These compounds should thus be taken into account when studying patients. Endogenous dicarbonyls are formed intracellularly, with an estimated daily production of methylglyoxal of ~120 µmol [14,47]. However, exogenous dicarbonyls, present in infusion fluids, peritoneal dialyses fluids, or diet, may contribute to plasma levels [15,48,49,50]. Certain interventions in critical illness, such as infusion fluids, tube feeding, continuous veno-venous hemofiltration or dialyses, extracorporeal membrane oxygenation, and mechanical ventilation, might affect exogenous dicarbonyls and endogenous metabolism. Therefore, medication use, exogenous sources of dicarbonyls, and critical care interventions should be considered when investigating dicarbonyl stress in critical care patients.

## 6. Study Population

Unfortunately, promising targets explored in laboratory experiments, such as inflammation, coagulation, endothelial dysfunction, and oxidative stress, have not yet led to novel therapeutics in critical care [4,7,8,9,10,11,12]. The most likely explanation for the contrasting results between laboratory and clinical results is heterogeneity in the study populations. As a matter of fact, heterogeneity has increasingly been recognized to hamper previous trials in critical illness and multi-organ failure [4,6,12,51]. Clinical studies may be heterogeneous in several ways: due to different underlying mechanisms of disease (i.e., inflammation versus infection); many different infectious agents; variation in genetic background; and differences in pre-clinical condition and comorbidity within the study population [4,6,51]. As critical care serves a heterogeneous population, strict selection criteria are key to reduce heterogeneity when investigating the role of increased dicarbonyl stress in multi-organ failure and persistent critical illness. This approach, however, limits generalizability of the results. Interestingly, methylglyoxal levels have been shown to be elevated in critical illness, independent of the septic focus, which is promising, although this study was relatively small [31]. As data in critical illness are scarce, and the role of heterogeneity with regard to dicarbonyl stress is unclear, new studies should start to carefully select a homogenous population, in order to provide more evidence in favour or against the hypothesis that dicarbonyls stress plays a role in multi-organ failure and persistent critical illness.

In conclusion, evidence suggests that increased glycolysis, hyperglycaemia, hypoxia, and inflammation, may increase dicarbonyl stress in critical illness; a mechanism that could lead to multi-organ failure and persistent critical illness. If so, several potential therapeutic interventions are possible. Therefore, this hypothesis requires further investigation.

## Abbreviations

AGEs	advanced glycation end products
CEL	*N*^ε^-(Carboxyethyl)lysine
CML	*N*^ε^-(Carboxymethyl)lysine
SOFA score	Sequential Organ Failure Assessment score
ACE	angiotensin converting enzyme
TNF	tumour necrosis factor
Nrf2	nuclear factor erythroid 2-related factor 2
